# Reply to Comment on ‘Necrotizing fasciitis in neonate by *Lichtheimia ramosa*: A case study’

**DOI:** 10.1099/acmi.0.000452

**Published:** 2022-09-13

**Authors:** Ashish William, Ravinder Kaur, Deepti Rawat, Neelam S. S. Kandir, Akanksha Sharma

**Affiliations:** ^1^​ Department of Microbiology, Lady Hardinge Medical College and Associated Hospitals, New Delhi, India

**Keywords:** mucormycosis, zygomycetes, *Lichtheimia ramosa*

Thank you for the comments. The responses for the above points are written below:

1. The isolation method was done phenotypically. The culture on Sabouraud's dextrose medium and white floccose growth on the medium after 24 hours of incubation. Lactophenol cotton blue mount showed the characteristic features of *Lichtheimia ramosa*.
2. The *Lichtheimia* species (*L. corymbifera* and *L. ramosa*) though very similar in morphology were differentiated phenotypically on the basis of macroscopic and microscopic appearance as given in the article. Features such as effuse growth, smooth, hyaline and more ellipsoidal sporangiospores are characterstic of *L. ramosa*. Therefore phenotypically (by microscopy from culture growth), it was identified as *L. ramosa* [1]. The molecular test for the sequencing whenever available should also be used to accurately aid in the speciation of fungi.
3. The two different samples were received post-debridment of cutaneous tissue after a period of 2 days which had shown similar growth of *Lichtheimia ramosa*.
4. The Amphotericin-B (1 mg/kg/day), Vancomycin (15 mg/kg/dose) and Colistin (2.5 mg/kg/dose) were given for a period of 1 week. After the surgical debridement and the identification of *Lichtheimia*, Amphotericin-B was continued which has been seen to show proper management of *Lichtheimia*.
